# Influence of microwave pretreatment on the total phenolics, antioxidant activity, moisture diffusivity, and rehydration rate of dried sweet cherry

**DOI:** 10.1002/fsn3.3703

**Published:** 2023-09-21

**Authors:** Fakhreddin Salehi, Moein Inanloodoghouz, Sara Ghazvineh

**Affiliations:** ^1^ Department of Food Science and Technology Bu‐Ali Sina University Hamedan Iran

**Keywords:** antioxidant capacity, Fick law, Gallic acid equivalents, Midilli equation, total phenolics content

## Abstract

The target of this work was to investigate the influence of microwave pretreatments (at five levels of 0, 30, 60, 90, and 120 s) on the total phenolics content, antioxidant potential, mass transfer rate, effective moisture diffusivity (*D*_eff_), and rehydration rate of sweet cherries (SC). The drying duration of microwave‐treated SC was shorter than the untreated sample. The average drying time of fresh SC microwaved for 0, 30, 60, 90, and 120 s were 220, 205, 190, 175, and 150 min, respectively. The *D*_eff_ values, total phenolics, and antioxidant capacity of microwave‐treated SC were higher than the untreated sample. In this study, the SC *D*_eff_ as determined by the second Fick law varied from 8.73 × 10^−10^ to 1.41 × 10^−9^ m^2^/s. The experimental data for the dehydration curves were fitted to different thin‐layer equations, and the Midilli equation using the experimental constants best described the drying rate of SC. As the microwave pretreatment time increased from 0 to 120 s, the total phenolics and antioxidant capacity of dried SC increased from 1491.4 μg Gallic acid equivalents (GAE)/g dry to 2272.1 μg GAE/g dry, and 54.47%–62.59% (*p* < .05). The microwave pretreatment enhanced the rehydration rate of dried SC. The rehydration percent of dried SC microwaved for 0, 30, 60, 90, and 120 s were 127.27%, 136.63%, 136.91%, 137.07%, and 136.72%, respectively.

## Graphical abstract

Microwave pretreatment not only increases drying rate and excellent energy efficiency, but also improves the retention of nutrients and total phenolics. Our results showed that the drying rate of sweet cherries during drying can be significantly improved by microwave pretreatment.

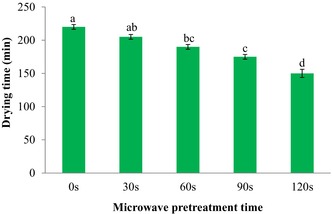

## 1 INTRODUCTION

Drying is a concurrent heat and mass transfer process that is widely used in fresh fruits and vegetables processing to prolong the shelf‐life of agricultural products by decreasing moisture content and lowering water activity (Salehi, 2023a, 2023b; Subramanyam et al., 2017). Microwave radiation is a type of non‐ionizing radiation energy utilized in foods production, which can efficiently alter the structural and functional properties of foods ingredients. The operating frequency is 915 MHz or 2450 MHz and is widely used in the food industries (Yılmaz & Tugrul, 2023). As a fast and effective heating source with together thermal and non‐thermal impacts, microwaves can directly affect the food material, thereby speeding upping physicochemical reactions, and drying rate, and producing high‐quality dried products (Mothibe et al., 2014; Wray & Ramaswamy, 2015; Xu, 2015). Also, microwave pretreatments can facilitate mass transfer in the dehydration procedure and reduce drying time for fruit and vegetable products (Mothibe et al., 2014; Sharma & Prasad, 2006; Wray & Ramaswamy, 2015). Yılmaz and Tugrul (2023) reported that microwave treatment is a useful technique due to short processing times (1–3 min).

The cultivation and consumption of sweet cherry (*Prunus avium* L.) has recently increased due to consumer awareness of their health benefits, as they are rich in polyphenolics (anthocyanins, phenolic acids, flavonoids, and hydroxycinnamic acid) (Blando & Oomah, 2019; Gonçalves et al., 2018; Salehi, Ghazvineh, & Inanloodoghouz, 2023; Wani et al., 2014). World production of SC has increased over the past 16 years, from 1.9 to 2.32 million tons, of which Turkey, United States of America, and Iran are the main producers (Blando & Oomah, 2019). Anthocyanins, responsible for the attractive color of SC, range from a few mg/100 g for pale SC to about 700 mg/100 g for dark SC (Wani et al., 2014). In addition, the total phenolic compounds and total anthocyanins present in SC range from 921 to 1468 μg GAE/g, and from 30.2 to 76.6 mg cyanidin‐3‐glucoside equivalents, respectively (Kim et al., 2005). The main anthocyanins present in dark SC are cyanidin‐3‐rutinoside (4–44 mg/100 g) and cyanidin‐3‐glucoside (2–243 mg/100 g), while hydroxycinnamate, neochlorogenic acid and p‐coumarylquinic was found in sufficient quantity (Kim et al., 2005; Wani et al., 2014). According to Prvulović et al. (2012), the total phenolics content of two sweet cherry varieties (Szomolyai Gombolyii and Valerij Cskalov) was 760.5 and 1109.6 μg GAE/g. SC are not only eaten raw, but also used to make jams, jellies, compotes, marmalades, syrups, and various drinks. SC are mainly eaten fresh and are also highly perishable (with a limited shelf life of 7–10 days) and drying is one method of preserving this fruit (Chockchaisawasdee et al., 2016; Doymaz & İsmail, 2011; Oancea et al., 2016; Salehi, Ghazvineh, & Inanloodoghouz, 2023; Vursavuş et al., 2006). Loss of firmness, color and flavor, discoloration of stems, drying and mold development limit SC shelf life in the long run (Wani et al., 2014).

SC contain high levels of nutritive and non‐nutritive compounds that are beneficial to human health (Chockchaisawasdee et al., 2016). Currently, microwave treatment is widely used in food products modification due to its benefits such as fast heating speed and high efficiency (Yılmaz & Tugrul, 2023; Zhang et al., 2022). We found no report on the impacts of microwave pretreatment on the dehydration rate, moisture diffusivity, and quality of SC in the literature. Therefore, the objective of this work was to examine the impacts of microwave pretreatment on the drying rate and moisture diffusivity of fresh SC, as well as on the total phenolic content, antioxidant capacity, and rehydration rate of air‐dried SC.

## 2 MATERIALS AND METHODS

### 2.1 Preparation of SC

SC were purchased from the market at Bahar, Hamedan Province, Iran. In this study, the water content (WC) of fresh and dried SC was calculated using an oven at 105°C for 5 h (Shimaz).

### 2.2 Microwave pretreatment

To apply the microwave pretreatment on the SC, a microwave oven (Gplus, Model; GMW‐M425S.MIS00; Goldiran Industries Co.) was used under atmospheric pressure. In this work, the influence of the microwave pretreatment time at five levels of 0, 30, 60, 90, and 120 s (power = 220 W) on the SC was examined.

### 2.3 Hot air drying

After each microwave pretreatment, the SC were dried in an oven (70 ± 2°C; Shimaz), until reaching a constant weight.

### 2.4 Drying rate

The dehydration kinetics of SC have been explained using 10 simplified drying equations (Manikantan et al., 2022; Salehi & Satorabi, 2021). In drying kinetics models, moisture ratio (MR) was employed to represent dehydration data, and for every experiment condition, MR values were plotted against drying time and models parameters were calculated and compared. Equation (1), calculate the MR of SC during drying:

(1) $$\mathrm{MR}=\frac{W_{t}-W_{e}}{W_{0}-W_{e}}$$

where *W*_t_, *W*_e_, and *W*_0_ are the WC of the SC at time *t*, equilibrium, and initial WC of the SC on a dry basis (g water/g dry matter). Data processing and modeling were performed using Matlab software (version R2012a) to estimate models parameters.

### 2.5 Calculation of moisture diffusivity (*D*_eff_)

Fick's second law of diffusion using spherical coordinates (Equation 2) was employed to calculate the moisture diffusivity of SC during hot air drying.

(2) $$\mathrm{MR}=\frac{M_{t}-M_{e}}{M_{0}-M_{e}}=\frac{6}{\pi^{2}}\sum_{n=1}^{\infty }\frac{1}{n^{2}}\exp\left[-\frac{\pi^{2}n^{2}D_{\mathrm{eff}}}{r^{2}}t\right]$$

where *D*_eff_ is the effective moisture diffusivity (m^2^/s), *r* is the average radius of the SC, *n* is positive integers and *t* is drying duration (s). For food dehydration process modeling, Equation (2) can be rewritten as Equation (3):

(3) $$\ln\mathrm{MR}=\ln\left(\frac{M_{t}-M_{e}}{M_{0}-M_{e}}\right)=\ln\left(\frac{6}{\pi^{2}}\right)-\left(\frac{\pi^{2}D_{\mathrm{eff}}t}{r^{2}}\right)$$

In this study, Equation (3) is employed for the calculation of *D*_eff_ values from the slope of lnMR and *t* (experimental drying duration, s) plot, which is calculated by Equation (4) (Salehi & Satorabi, 2021):

(4) $$\text{Slope}=\frac{\pi^{2}D_{\mathrm{eff}}}{r^{2}}$$

### 2.6 Determination of total phenolics content and antioxidant capacity

The extraction of phenolic compounds from SC was performed according to the method described by Salehi, Ghazvineh, and Inanloodoghouz (2023). In addition, the total phenolics content and antioxidant capacity were determined according to the method described by Salehi, Ghazvineh, and Inanloodoghouz (2023). The Folin–Ciocalteu (Folin–Ciocalteu's phenolics reagent; Sigma‐Aldrich) method was followed for measuring the total phenolics content of dried SC. The absorbance of samples (765 nm, UV–VIS spectrophotometer, XD‐7500; Lovibond) was compared with the Gallic acid standard curve (*R* ^2^ = 9998). The results were expressed as μg GAE/g dry matter. For the analysis of the antioxidant capacity, 2,2‐Diphenyl‐1‐picrylhydrazyl (DPPH; Sigma‐Aldrich) free radical scavenging activity (FRSA) method was used according to Salehi, Ghazvineh, and Inanloodoghouz (2023).

### 2.7 Rehydration

A high rehydration capacity value indicates a high‐quality dried sample. This means that higher rehydration capacity means less tissue and structural damage (Alvi et al., 2023). The rehydration tests were conducted with a water bath (R.J42; Pars Azma Co.). Dried SC were weighed and immersed for 30 min in distillated water in a 200 mL glass beaker at 50°C. Then, the extra moisture was drained for 30 s and the samples were weighed again. The rehydration ratio values (%) of dried SC were determined as the ratio of the final weight of rehydrated SC over the dried SC weight × 100 (Salehi, Razavi Kamran, & Goharpour, 2023).

### 2.8 Statistical analysis

An analysis of variance (ANOVA) was conducted to analyze drying rate and physicochemical responses of SC according to the time of microwave exposure by SPSS 21 software (IBM). A post hoc test by Duncan's multiple range calculation at *p*‐value < .05 was preceded for ANOVA results. When a significant difference was detected (*p* < .05), all these data were adopted a one‐way ANOVA to determine differences in measured variables among experimental groups. Each treatment was conducted in triplicate and the results are expressed as the mean ± standard deviation (mean ± SD, *n* = 3).

## 3 RESULTS AND DISCUSSION

### 3.1 Drying rate

**FIGURE 1:**
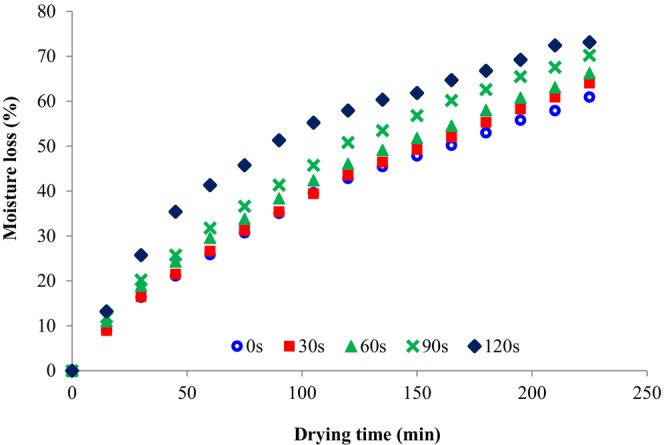
Moisture loss of microwave‐treated sweet cherries during drying in the hot air dryer.

Dried fruits have a long shelf‐life, making them a good substitute for fresh fruits and allowing you to have off‐season fruits available. Hot air drying is the most common foods preservation method for fruits and vegetables (Salehi, 2021). The impacts of microwave pretreatment on the moisture loss of SC during dehydration in the hot air dryer are shown in Figure 1. The moisture loss rate of treated SC was higher than the untreated SC. As seen in this figure, the microwave pretreatment enhanced the rate at which water escaped from the samples and as a result, it increased the dehydration rate of the SC.

**FIGURE 2:**
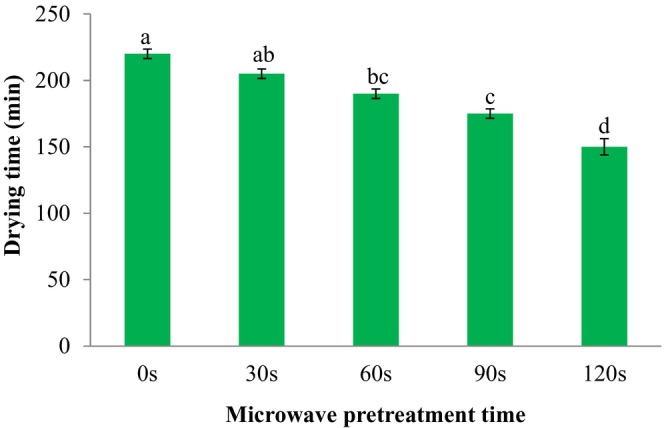
Effect of microwave pretreatment on the drying time of sweet cherries. Data are shown as mean ± standard deviation (*N* = 3). According to the one‐way ANOVA and the Duncan post hoc test, different letters above the columns indicate a significant differences (*p* < .05).

Microwave drying not only provides high drying speeds and excellent energy efficiency, but also improves nutrient and color retention (Alvi et al., 2023). Changes in drying time during hot air drying of control and microwave‐pretreated SC are reported in Figure 2. In this figure, the various letters above the columns indicate significant differences at *p* < .05 level between pretreatments (the time of microwave exposure). The drying duration of treated SC was shorter than the untreated samples. As the microwave pretreatment time enhanced from 0 to 120 s, the dehydration duration of SC in the hot air dryer significantly decreased from 220 to 150 min (*p* < .05). Sharma and Prasad (2006) used a laboratory microwave for drying of garlic. They confirmed that the microwave drying of garlic cloves decreased drying duration compared to hot air dehydration, and the quality attributes of dehydrated product by microwave methods, was found higher.

### 3.2 Effective moisture diffusivity coefficient (*D*_eff_)

**FIGURE 3:**
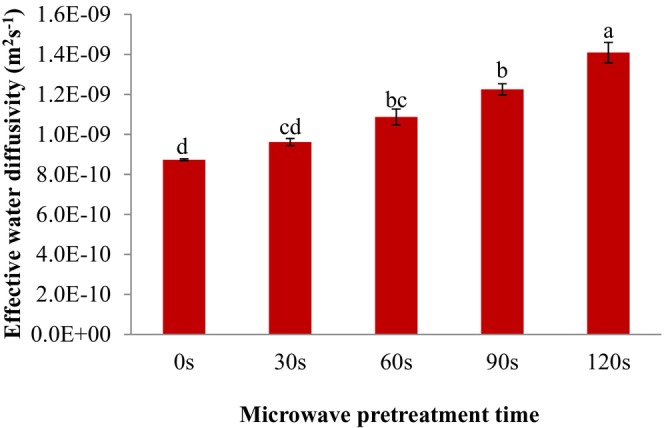
Effect of microwave pretreatment on the effective water diffusivity coefficient of sweet cherries. Data are shown as mean ± standard deviation (*N* = 3). According to the one‐way ANOVA and the Duncan post hoc test, different letters above the columns indicate a significant differences (*p* < .05).

Moisture content promotes the growth of microorganisms and is a major cause of food spoilage. Therefore, decreasing the moisture content economically reduces foods spoilage (Salehi, 2021; Subramanyam et al., 2017). Microwave drying transfers moisture faster than traditional drying methods and better preserves the nutritional value of food ingredients (Aydar et al., 2022). The impacts of microwave pretreatment on the *D*_eff_ values of SC are shown in Figure 3. The *D*_eff_ values of treated SC were higher than the untreated sample. As the microwave pretreatment time increased from 0 to 120 s, the *D*_eff_ values of SC in the hot air dryer increased from 8.73 × 10^−10^ to 1.41 × 10^−9^ m^2^/s (*p* < .05). The purpose of the study by Simsek and Süfer (2021a) was to examine the impacts of various pretreatment methods on the refractance window dehydration, color kinetics and bioactive content of white SC. Their results showed that the minimum drying time (180 min) and the maximum *D*_eff_ value (2.4 × 10^−9^ m^2^/s) were obtained with freezing pretreatment, respectively. In another study, Simsek and Süfer (2021b) examined the impacts of various pretreatments on combined hot air and microwave hot air drying of white SC. Their results confirm that the *D*_eff_ value of white SC ranges from 1.7 × 10^−10^ to 5.2 × 10^−10^ m^2^/s in hot air drying and from 4.3 × 10^−10^ to 1.8 × 10^−9^ m^2^/s in microwave hot air drying.

### 3.3 Kinetics modeling

**FIGURE 4:**
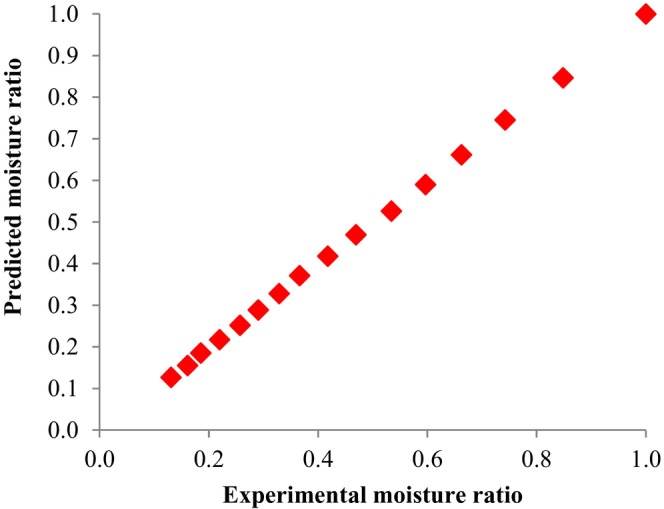
Comparison of fitted data by Midilli model with experimental results of moisture ratio (microwave pretreatment time = 90 s).

The drying behavior of untreated and microwave‐treated SC in the hot air dryer was fitted with the Midilli model (Salehi, Ghazvineh, & Inanloodoghouz, 2023). This model showed a good fit with the maximum *r*‐value (higher than 0.9987) and the minimum SSE (sum of squared errors), and RMSE (root mean squared error) values (lower than 0.0028 and 0.0154, respectively) for all conditions compared to that of the other models. The calculated constant coefficients of the Midilli equation include *a*, *k*, *n*, and *b*, are reported in Table 1 along with matching statistical error values (SSE, RMSE, and *r*) for all dehydration conditions. Mean values of SSE, RMSE, and *r* for all experiments ranged from 0.0002 to 0.0028, 0.0039 to 0.0154, and 0.9987 to 0.9999, respectively. Figure 4 demonstrates the comparison of fitted moisture ratio data using the Midilli equation with experimental results. The results show that the Midilli equation is suitable for describing the drying kinetics of untreated and microwave‐treated SC.

**TABLE 1:** The constants and coefficients of the approved model (Midilli).

Microwave pretreatment time (s)	*a*	*k*	*n*	*b*	SSE	*r*	RMSE
0	1.0015^a^	0.0144	0.7774	−0.0007	0.0008	.9995	0.0082
30	1.0020	0.0174	0.7453	−0.0009	0.0005	.9997	0.0065
60	1.0003	0.0214	0.7104	−0.0010	0.0005	.9997	0.0065
90	0.9998	0.0168	0.7871	−0.0009	0.0003	.9998	0.0051
120	1.0040	0.0236	0.8208	−0.0003	0.0016	.9993	0.0111

^a^ Mean (*N* = 3).

### 3.4 Total phenolics content

**FIGURE 5:**
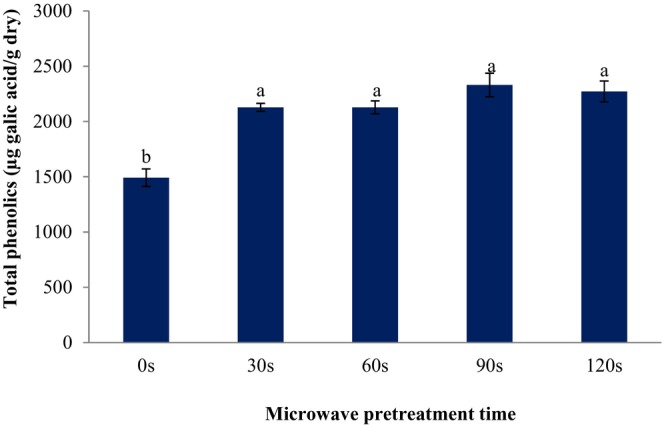
Effect of microwave pretreatment on the total phenolics of dried sweet cherries. Data are shown as mean ± standard deviation (*N* = 3). According to the one‐way ANOVA and the Duncan post hoc test, different letters above the columns indicate a significant differences (*p* < .05).

SC are rich in phenolic components. Phenolic compounds are one of the major groups that acts as an essential antioxidant or free radical terminator. Measurement of total phenolics is one of the important criteria for estimating the antioxidant capacity of a sample (Sadiq et al., 2023). In this study, the total phenolics of fresh SC was 5176.2 μg GAE/g dry. The impact of microwave pretreatment on the total phenolics of SC are shown in Figure 5. The total phenolics of microwave‐treated SC was higher than the untreated samples. In this figure, the various letters above the columns indicate significant differences at *p* < .05 level between pretreatments (the time of microwave exposure). The average total phenolics of dried SC microwaved for 0, 30, 60, 90, and 120 s were 1491.4, 2127.8, 2127.2, 2330.7, and 2272.1 μg GAE/g dry, respectively. Cheng et al. (2019) suggested that microwave vacuum drying is the most suitable drying method to preserve the total phenolics, antioxidant activity, and bioactivity of green coffee beans. The results of Simsek and Süfer (2021a) confirmed that the freezing pretreatment improved the total phenolic content (4180 ± 0.86 μg GAE/g dry matter) and antioxidant capacity of dried white SC, as compared to control and other pretreatment methods. In another study, Simsek and Süfer (2021b) examined the impacts of various pretreatments on combined hot air and hot air‐microwave drying of white SC. Their results confirmed that the total phenolic content of freezing pretreated and hot air dried at 50°C and hot air dried at 70°C control samples were 1481 and 6181 μg GAE/g, respectively.

### 3.5 Antioxidant capacity

**FIGURE 6:**
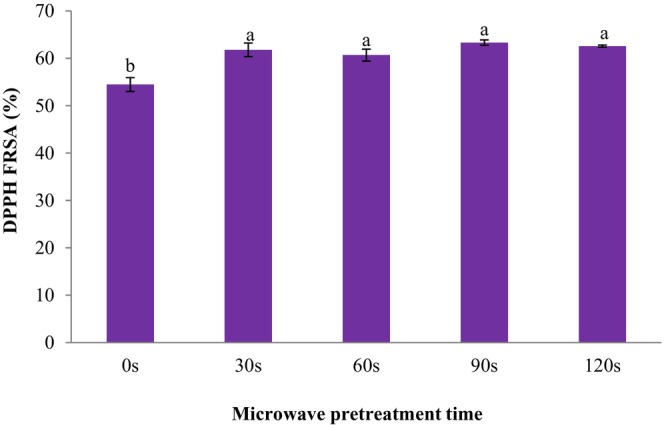
Effect of microwave pretreatment on the 2,2‐Diphenyl‐1‐picrylhydrazyl (DPPH) free radical scavenging activity (FRSA) of dried sweet cherries. Data are shown as mean ± standard deviation (*N* = 3). According to the one‐way ANOVA and the Duncan post hoc test, different letters above the columns indicate a significant differences (*p* < .05).

Consumer demand for SC is increasing due to their sweet taste, attractive color, and high antioxidant content (Wani et al., 2014). Simsek and Süfer (2021a) results confirmed the need to use various assays to analyze the antioxidant capacity of SC, since the specific antioxidant capacity of different compounds has can be detected by different assays based on various mechanisms. The DPPH method is based on the measurement of the FRSA of antioxidant compounds. The impact of microwave pretreatment on the DPPH FRSA of SC is shown in Figure 6. In this figure, the various letters above the columns indicate significant differences at *p* < .05 level between pretreatments (the time of microwave exposure). The antioxidant properties of the dried SC were retained by the microwave pretreatment. The antioxidant capacity of treated SC was higher than the untreated sample. The average DPPH FRSA of dried SC microwaved for 0, 30, 60, 90, and 120 s were 54.47%, 61.80%, 60.68%, 63.32%, and 62.59%, respectively. The impacts of edible coating and sonication on the total phenolic content and antioxidant capacity of SC were examined by Salehi, Ghazvineh, and Inanloodoghouz (2023). The authors reported that the DPPH FRSA of uncoated and coated SC ranged from 39.75% to 61.04%.

### 3.6 Rehydration

**FIGURE 7:**
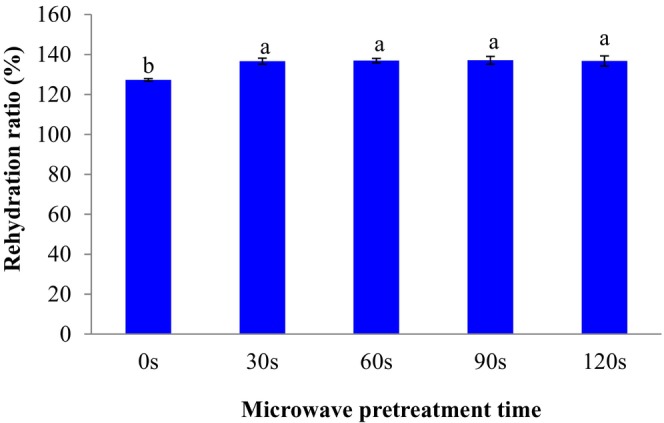
Effect of microwave pretreatment on the rehydration ratio of dried sweet cherries. Data are shown as mean ± standard deviation (*N* = 3). According to the one‐way ANOVA and the Duncan post hoc test, different letters above the columns indicate a significant differences (*p* < .05).

The impacts of microwave pretreatment on the rehydration percent of dried SC are shown in Figure 7. The rehydration rate of treated samples by microwave was considerably higher than the untreated sample (*p* < .05). This issue can be due to higher volume and porous structure with lower shrinkage in microwave‐treated samples which give higher diffusion of moisture inside the dried SC cells, and therefore, higher rehydration ratio. The average rehydration ratio of dried SC microwaved for 0, 30, 60, 90, and 120 s were 127.27%, 136.63%, 136.91%, 137.07%, and 136.72%, respectively. Nayi et al. (2023) reported that rehydrated sweet corn obtained from samples treated by microwave‐blanching and drying at 70°C had high maintenance of total sugars, ascorbic acid, geometric mean diameter, and color. The impacts of edible coating and sonication on the rehydration ratio of SC were examined by Salehi, Ghazvineh, and Inanloodoghouz (2023). The authors reported that the rehydration ratio of uncoated and coated SC ranged from 141.8% to 176.2%.

## 4 CONCLUSION

In this work, the impacts of microwave pretreatment on the total phenolics content, antioxidant potential, and thin‐layer drying rates of SC were studied. The drying duration of microwave‐treated SC was shorter than the untreated sample. The *D*_eff_ values of microwave‐treated SC were higher than the untreated sample. The drying behavior of untreated and treated SC was fitted with the Midilli equation. This equation showed a good fit with the maximum *r*‐value and the minimum SSE, and RMSE values for all conditions compared to that of the other models. The total phenolics of microwave‐treated SC was higher than the untreated sample. Also, the antioxidant potential of the dried SC was retained by the microwave pretreatment. The rehydration rate of treated SC by microwave was considerably higher than the untreated sample (*p* < .05). This may be due to less structural collapse during drying of microwaved SC, which may be due to the shorter heating time of microwaved SC. Considering the drying time, *D*_eff_ values, total phenolics content, antioxidant activity, and rehydration ratio, microwave pretreatment will be more promising for SC pretreatment before drying process.

## AUTHOR CONTRIBUTIONS

**Fakhreddin Salehi:** Conceptualization (lead); data curation (equal); formal analysis (equal); investigation (lead); software (equal); supervision (lead); writing – original draft (equal); writing – review and editing (equal). **Moein Inanloodoghouz:** Data curation (equal); formal analysis (equal); software (equal). **Sara Ghazvineh:** Data curation (equal); formal analysis (equal); software (equal).

## CONFLICT OF INTEREST STATEMENT

None.

## Data Availability

All data generated or analyzed during this study are included in this published article.
